# Effect of Smartphone-Enabled Health Monitoring Devices vs Regular Follow-up on Blood Pressure Control Among Patients After Myocardial Infarction: A Randomized Clinical Trial

**DOI:** 10.1001/jamanetworkopen.2020.2165

**Published:** 2020-04-16

**Authors:** Roderick W. Treskes, Loes A. M. van Winden, Nicole van Keulen, Enno T. van der Velde, Saskia L. M. A. Beeres, Douwe E. Atsma, Martin Jan Schalij

**Affiliations:** 1 Department of Cardiology, Leiden University Medical Center, Leiden, the Netherlands

## Abstract

**Importance:**

Smart technology via smartphone-compatible devices might improve blood pressure (BP) regulation in patients after myocardial infarction.

**Objectives:**

To investigate whether smart technology in clinical practice can improve BP regulation and to evaluate the feasibility of such an intervention.

**Design, Setting, and Participants:**

This study was an investigator-initiated, single-center, nonblinded, feasibility, randomized clinical trial conducted at the Department of Cardiology of the Leiden University Medical Center between May 2016 and December 2018. Two hundred patients, who were admitted with either ST-segment elevation myocardial infarction or non–ST-segment acute coronary syndrome, were randomized in a 1:1 fashion between follow-up groups using smart technology and regular care. Statistical analysis was performed from January 2019 to March 2019.

**Interventions:**

For patients randomized to regular care, 4 physical outpatient clinic visits were scheduled in the year following the initial event. In the intervention group, patients were given 4 smartphone-compatible devices (weight scale, BP monitor, rhythm monitor, and step counter). In addition, 2 in-person outpatient clinic visits were replaced by electronic visits.

**Main Outcomes and Measures:**

The primary outcome was BP control. Secondary outcomes, as a parameter of feasibility, included patient satisfaction (general questionnaire and smart technology–specific questionnaire), measurement adherence, all-cause mortality, and hospitalizations for nonfatal adverse cardiac events.

**Results:**

In total, 200 patients (median age, 59.7 years [interquartile range, 52.9-65.6 years]; 156 men [78%]) were included, of whom 100 were randomized to the intervention group and 100 to the control group. After 1 year, 79% of patients in the intervention group had controlled BP vs 76% of patients in the control group (*P* = .64). General satisfaction with care was the same between groups (mean [SD] scores, 82.6 [14.1] vs 82.0 [15.1]; *P* = .88). The all-cause mortality rate was 2% in both groups (*P* > .99). A total of 20 hospitalizations for nonfatal adverse cardiac events occurred (8 in the intervention group and 12 in the control group). Of all patients, 32% sent in measurements each week, with 63% sending data for more than 80% of the weeks they participated in the trial. In the intervention group only, 90.3% of patients were satisfied with the smart technology intervention.

**Conclusions and Relevance:**

These findings suggest that smart technology yields similar percentages of patients with regulated BP compared with the standard of care. Such an intervention is feasible in clinical practice and is accepted by patients. More research is mandatory to improve patient selection of such an intervention.

**Trial Registration:**

ClinicalTrials.gov Identifier: NCT02976376

## Key Points

**Question:**

Does the use of smartphone technology have an effect on improved blood pressure regulation in patients after myocardial infarction and is it feasible to implement?

**Findings:**

This randomized clinical trial of 200 patients found that there was no statistically significant difference in regulated blood pressure between groups at 12 months. Exploratory secondary outcomes showed that clinical outcomes and patient satisfaction were similar between groups.

**Meaning:**

The use of smart technology in the follow-up of patients with myocardial infarction did not have an effect on improved blood pressure control, but implementing such an intervention was feasible and accepted by patients.

## Teaser

This randomized clinical trial investigates whether smart technology via smartphone-compatible devices improves blood pressure regulation in patients after myocardial infarction.

## Introduction

Pharmaceutical treatment and implementation of cardiac rehabilitation programs have improved the prognosis of patients with acute myocardial infarction (AMI).^1^ To achieve maximum benefit, compliance with medication intake and cardiac rehabilitation are essential for decreasing the risk of recurrent AMI, heart failure, and cardiovascular mortality. A recent study^2^ showed that nonadherence to β-blockers, angiotensin-converting enzyme inhibitors, and statins was associated with an increase in cardiovascular-associated and all-cause mortality in patients with coronary artery disease. Other studies^3^ have shown that patients who are better informed are more adherent to therapy. Therefore, there is a need for an intervention that may help to improve the compliance of patients with guideline-based therapy.

Broadly speaking, the term “eHealth” refers to the delivery of medicine using information technology and has been suggested as a tool to deliver health care.^4,5^ It can be delivered using personal computers, mobile phones, or tablets. One advantage of delivering health care via these mobile devices is that an already existing infrastructure can be used. Currently, the majority of the population in the Western world has internet access and is in possession of a smartphone.^6^ Recently, it was shown that 92% of the Dutch population (aged ≥12 years) uses the internet, and 89% of the population owns a smartphone.^7^

One marker that is specifically associated with compliance with guideline-based therapy is blood pressure (BP).^2,8^ Therefore, a feasibility randomized clinical trial (RCT) investigating the effect of smart technology on BP is presented here. Secondary outcomes included patient satisfaction and hospitalization for adverse cardiac events as markers of feasibility.

## Methods

### Trial Conduct

The Box was an open-label, single-center, parallel-group, feasibility RCT, conducted at the Department of Cardiology of the Leiden University Medical Center in Leiden, the Netherlands, between 2016 and 2018. A detailed description of the methods has been published previously.^9^ Patients were randomized in a 1:1 fashion between a smart technology intervention (“The Box”) and regular follow-up. The complete trial protocol is shown in Supplement 1.

Regular follow-up was defined as 4 visits to the outpatient clinic (1, 3, 6, and 12 months after AMI). A detailed version of this protocol has been published previously.^10^ For the outpatient clinic, patients had to come to the hospital. Each outpatient clinic consisted of a 10-second 12-lead electrocardiogram (ECG), a BP measurement by a nurse practitioner, and a 15-minute patient interview by a nurse practitioner. The 1-month, 6-month, and 12-month visits included laboratory testing. At 3 months, a stress echocardiogram was performed. At 3 and 6 months, a 24-hour Holter monitoring procedure was performed. At 6 and 12 months, a transthoracic echocardiogram was performed.

The protocol of this trial was approved by the Medical Ethics Committee of the Leiden University Medical Center. The trial was conducted in accordance with the principles of the Declaration of Helsinki^11^ and Good Clinical Practice.^12^ All participants provided written offline informed consent before participation. Devices used in this study are all Conformité Européene–marked. This study follows the Consolidated Standards of Reporting Trials (CONSORT) reporting guideline.

### Intervention Group

In the intervention group, the 1-month and 6-month follow-up visits were replaced by an electronic visit (e-visit). This e-visit consisted of a patient interview done via a secured video connection. Patients used a tablet, smartphone, or computer. Via internet, both nurse practitioner and patients logged into a virtual meeting room. Patients could log in from anywhere (including home, the workplace, or a holiday destination), as long as they had a stable internet connection. Consequently, patients did not have to come to the hospital. The e-visits were performed by the same nurse practitioner as the regular outpatient clinic visits. The content of the patient interview was comparable to that of the regular outpatient clinic visit.

### The Box

The smart technology intervention included 4 smartphone-compatible devices: a BP monitor (Wireless Blood Pressure Monitor; Withings), a step counter (Pulse Ox; Withings), a weight scale (Smart Body Scale Analyzer; Withings), and a single-lead ECG device (Kardia; AliveCor Inc). The BP monitor is an oscillometric device. It is a preformed cuff that has to be applied to the bare upper arm. It communicates with the device-dedicated application (app) on the smartphone via Bluetooth. The inflation of the cuff has to be started in the app. Patients have to sit still with their upper arm on the same height as their chest. The measurement result is displayed in the app. As such, patients immediately can see their own BPs. Data are automatically transferred to and integrated in the department’s electronic medical record. As such, results are available to any physician who is legally allowed to view an individual patient’s electronic medical record. Devices have to be installed and synchronized the first time they are used. The step counter is a wristwatch that records the time and number of steps per day. These data, as well as data acquired by the weight scale, are sent to the app via Bluetooth.

The single-lead ECG device is approximately the size of a credit card. It has 2 electrodes. To record a single-lead ECG, a patient has to position 2 fingers of the right hand to the right electrode and 2 fingers of the left hand to the left electrode. An ultrasound signal is generated and converted into an electrical signal in the smartphone, which is displayed as single-lead ECG on the smartphone screen. The ECG readings were sent to the hospital, where they were checked by a project-dedicated health care professional with ample training, supervised by a consultant cardiologist.

Patients were asked to record their steps continuously, their BP and weight daily, and their ECG daily and to record symptoms of possible cardiac origin. Data were reviewed daily by a project-dedicated professional with ample training. Data were not continuously monitored. Therefore, the smart technology intervention was not a substitute for emergency care. Patients were contacted in case their systolic BP exceeded 139 mm Hg or diastolic BP exceeded 89 mm Hg. Patients were also contacted in case of newly diagnosed arrhythmias or at least 4 newly diagnosed symptomatic premature ventricular contractions on the single-lead ECG. All clinical data were stored in the departmental electronic patient files (EPD-Vision; Leiden University Medical Center).

### Patient Population

Patients admitted to the Department of Cardiology of the Leiden University Medical Center with AMI were eligible for participation. Patients were excluded if they were younger than 18 years, pregnant, unwilling to sign informed consent, or unable to speak Dutch or English.

### Outcomes

The primary end point of the trial was proportion of patients with controlled BP after 1 year of follow-up. Controlled BP was defined as a systolic BP less than or equal to 139 mm Hg and a diastolic BP less than or equal to 89 mm Hg. Blood pressure was measured at the fourth outpatient clinic visit by a nurse practitioner with a handheld aneroid sphygmomanometer, which was placed around the bare upper arm of patient’s preference.

### Patient Satisfaction (Secondary Outcome)

Patient satisfaction was measured using the Patient’s Satisfaction Questionnaire.^13^ This questionnaire has been validated to measure satisfaction of care of 7 domains: access to care, financial aspects, availability of resources, continuity of care, technical quality, interpersonal manner, and general satisfaction. Patients read 68 statements regarding health care and indicated whether they (strongly) agree, (strongly) disagree, or neither agree nor disagree. Each answer was given a score, which was converted into a final score between 0 and 100. A score close to 100 indicated high patient satisfaction. Questionnaires were given to patients 1, 6, and 12 months after inclusion.

### Patient’s Acceptance and Measurement Adherence (Intervention Group Only)

An exploratory nonvalidated questionnaire was given to the intervention group after 12 months. This questionnaire included questions on satisfaction regarding the intervention in general, the individual devices, and the outpatient e-visits. A question started with (“Are you satisfied with ….”), to which patients could answer with either yes or no. Percentages of patients who answered yes were taken as measurement of patient’s acceptance of the smart technology intervention. If a patient sent in either a BP or a single-lead ECG, he or she was considered adherent for that week.

### Participation Rate

During the trial, the numbers of patients who refused to participate and their primary reasons for not participating were recorded in the database. These reasons were classified as follows: the fear that they would be confronted with their disease too often, fear of not being able to cope with technology, wanting to be followed-up in a different hospital, and refusing to give a reason or another reason for not participating.

### Mortality

All-cause mortality was counted if a patient was pronounced dead by a licensed physician and registered as deceased in the municipality register. Mortality was considered a cardiovascular death if the primary cause of death was a cardiovascular event (eg, AMI, hospitalization for decompensated heart failure, or ventricular tachycardia in ischemic cardiomyopathy).

### Hospitalizations for Nonfatal Cardiac Adverse Events

As an exploratory secondary end point for safety, hospitalizations for nonfatal cardiac adverse events were registered. These events were assessed by a clinical end point committee, consisting of 2 senior independent cardiologists who were blinded to patient data and treatment allocation. In case of disagreement, a third cardiologist was involved to reach a decision. Definitions are given in the eAppendix in Supplement 2.

### Statistical Analysis

A power calculation was performed in R statistical software version 3.2.0 for Windows (R Project for Statistical Computing). A comparison of 2 proportions is used. It was expected that 95% of patients in the intervention group would have regulated BP and that 75% of patients in the control group would have regulated BP. An α of 0.05, a β of 0.20, and a margin of 0.07 were chosen, yielding a sample size of 200 patients.

Analyses of primary and secondary end points were performed using SPSS statistical software version 23.0 (IBM Corp). Data were analyzed according to the intention-to-treat principle. Continuous variables are summarized as mean and SD. Differences in continuous variables were tested for significance with a Mann-Whitney *U* test. The primary end point was tested for significance with a χ^2^ test. Differences in hospitalizations for nonfatal adverse cardiac events were tested for significance with a Fisher exact test. All tests were 2-sided. An α ≤.05 was considered statistically significant. Statistical analysis was performed from January 2019 to March 2019.

## Results

### Patients

**Figure 1.**
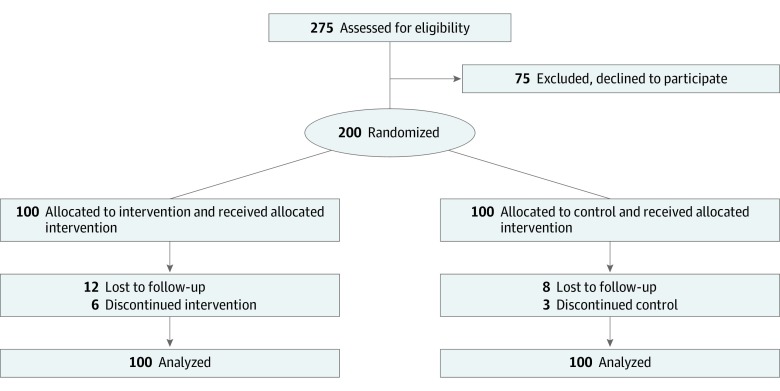
CONSORT Flowchart

In total, 200 patients (median age, 59.7 years [interquartile range {IQR}, 52.9-65.6 years]; 156 men [78%]; median body mass index [calculated as weight in kilograms divided by height in meters squared], 27.1 [IQR, 24.8-30.1]) were included, of whom 100 were randomized to the intervention group and 100 to the control group. There were no substantial differences in baseline characteristics between the intervention group and the control group (median age, 60.1 years [IQR, 52.7-66.3 years] vs 59.1 years [IQR, 53.1-65.0 years]; median body mass index, 27.1 [IQR, 24.8-30.1] vs 27.1 [IQR, 24.5-30.3]; 40% vs 37% of patients with hypertension) (Table 1). A CONSORT flowchart of analyzed patients is shown in Figure 1.

**Table 1.** Baseline Characteristics of the Population

Characteristic	Participants, No. (%)		
	Total (N = 200)	Intervention (n = 100)	Control (n = 100)
Age, median (IQR) [range], y	59.7 (52.9-65.6) [28.4-81.0]	60.1 (52.7-66.3) [37.6-81.0]	59.1 (53.1-65.0) [28.4-79.6]
Male	156 (78)	81 (81)	75 (75)
Body mass index, median (IQR)^a^	27.1 (24.8-30.1)	27.1 (24.8-30.1)	27.1 (24.5-30.3)
Maximum troponin level, median (IQR), μg/L	2.5 (0.8-5.6)	2.5 (0.8-5.4)	2.4 (0.8-5.6)
Maximum creatine kinase level, median (IQR), U/L	973 (401-2047)	973 (454-2037)	998 (309-2070)
ST-elevation myocardial infarction	157 (79)	79 (79)	78 (78)
Hypertension	77 (39)	40 (40)	37 (37)
Chronic obstructive pulmonary disease	10 (5)	6 (6)	4 (4)
Type 2 diabetes	23 (12)	12 (12)	11 (11)
Previous cardiovascular incidents			
Myocardial infarction	17 (9)	8 (8)	9 (9)
Stroke	5 (3)	4 (4)	1 (1)
Transient ischemic attack	8 (4)	6 (6)	2 (2)
Known electrocardiogram abnormalities	21 (11)	10 (10)	11 (11)
Smartphone use every day	176 (88)	89 (89)	87 (87)
Previous home monitoring	45 (23)	25 (25)	20 (20)

Abbreviation: IQR, interquartile range.

To convert creatine kinase to microkatals per liter, multiply by 0.0167

^a^ Body mass index is calculated as weight in kilograms divided by height in meters squared.

Of the 200 patients included in the study, 24 did not reach the 1-year follow-up. Four patients died: 2 in the intervention group and 2 in the control group. Of these 4 deaths, 3 had a cardiac cause (1 in the intervention group and 2 in the control group). One patient died of alcohol intoxication. The all-cause mortality rate was 2% in both groups (*P* > .99).

Twenty patients were lost to follow-up (12 in the intervention group and 8 in the control group). However, according to the municipality registration, all were alive at 12 months after AMI.

### Controlled BP

In intervention group, 79% of patients had regulated BP at 12 months. In the control group, 76% of patients had a regulated BP. This difference was not statistically significant (*P* = .64).

### Patient Satisfaction

Equal outcomes were observed in the Patient’s Satisfaction Questionnaire. Mean (SD) scores for general satisfaction were 82.6 (14.1) in the intervention group and 82.0 (15.1) in the control group (*P* = .88). Other high-scoring domains were interpersonal (mean [SD] scores, 86.9 [13.2] for intervention vs 86.9 [14.4] for control), communication (mean [SD] scores, 83.4 [13.5] for intervention vs 85.2 [14.6] for control), and technical quality (mean [SD] scores, 83.6 [12.4] for intervention vs 82.8 [13.7] for control). All scores and *P* values are given in Table 2. No differences between the intervention and control groups were statistically significant.

**Table 2.** Domain Scores of Patient Satisfaction

Domain	Score, mean (SD)		*P* value
	Intervention group	Control group	
Access	82.5 (11.7)	81.5 (13.1)	.65
Technical quality	83.6 (12.4)	82.8 (13.7)	.77
Communication	83.4 (13.5)	85.2 (14.6)	.50
Interpersonal	86.9 (13.2)	86.9 (14.4)	.72
Time	81.3 (19.9)	82.2 (19.6)	.58
General satisfaction	82.6 (14.1)	82.0 (15.1)	.88

### Patient’s Acceptance (Intervention Group Only)

Of all patients in the intervention group, 90% indicated that they were satisfied with the smart technology intervention. Satisfaction with individual devices was 88% for the BP monitor, 88% for the weight scale, 4% for the step counter, and 89% for the ECG device. A total of 80% of patients were satisfied with the e-visit. Patients who were not satisfied named technical problems as primary reason for their dissatisfaction. Finally, 93% indicated that they appreciated the extra checkup by the hospital, and 96% indicated that they appreciated that they could view their own health data.

### Adherence

Of all patients who finished the intervention, 32% sent measurements each week. In total, 63% sent measurements in more than 80% of all 52 weeks they participated in the trial.

### Participation Rate

In total, 275 patients were approached for participation. The trial had a participation rate of 73%. These patients had a mean age of 65 years and 64% were male. The main reason (40%) to refuse participation in the trial was because patients feared they would be confronted with their disease too often. Other reasons included fear of the technology (33%), wanting to be followed up in a different hospital (8%), no reason (4%), or another reason not to participate (15%).

### Hospitalizations for Nonfatal Adverse Cardiac Events

**Figure 2.**
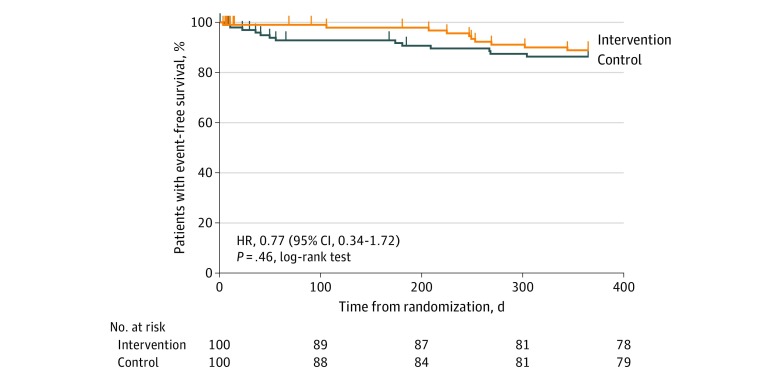
Kaplan-Meier Curve for Event-Free Survival in the Intervention and Control Groups HR indicates hazard ratio.

In total, 20 hospitalizations for nonfatal adverse cardiac events occurred. Eight occurred (2 recurrent AMIs, 2 out-of-hospital cardiac arrests, and 4 elective revascularizations) in the intervention group and 12 (1 heart failure admission, 2 recurrent AMIs, and 9 elective revascularizations) in the control group (Table 3). These differences were not statistically significant. A Kaplan-Meier curve showing event-free survival is shown in Figure 2.

**Table 3.** Mortality and Hospitalizations for Nonfatal Adverse Cardiac Events

Variable	Participants, No. (%)		*P* value
	Intervention group (n = 100)	Control group (n = 100)	
All-cause mortality	2 (2)	2 (2)	>.99
Recurrent myocardial infarction	2 (2)	2 (2)	.62
Hospitalization for heart failure	0	1 (1)	>.99
Elective revascularization	4 (4)	9 (9)	.57
Out-of-hospital cardiac arrest	2 (2)	0	.50

## Discussion

This study reports the results of an exploratory RCT comparing smart technology–enabled follow-up with usual care for control of BP after AMI. The key findings are that the percentage of patients with regulated BP did not differ between the intervention and control group, the percentage of hospitalizations was similar in both groups, and patient satisfaction scores were similar.

Trials of remote monitoring were already conducted in the 1980s, with telemonitoring of symptoms via the telephone.^14^ Since the introduction of the Apple iPhone in 2007, the number of scientific articles about telemonitoring has increased each year.^15^ Several RCTs have evaluated the use of smart technology in the follow-up of patients with AMI. These trials predominantly use smart technology for telerehabilitation.^16,17^ One trial^16^ found a morbidity benefit associated with the use of smartphone technology in the rehabilitation setting, with a reduction in days lost to cardiovascular rehospitalizations. There is cumulative evidence showing that telerehabilitation is effective for patients after AMI.^16,17^ In general, the follow-up of patients after AMI is performed in an outpatient clinic by a cardiologist or specialized nurse. To our knowledge, no trial has yet compared the use of eHealth in the outpatient clinic for patients after AMI. To our knowledge this is the first trial to partially substitute physical outpatient clinic visits by e-visits in this patient population. Electronic visits can reduce time and costs for patients and can lower overhead costs. Therefore, this trial adds to the existing literature that eHealth in the outpatient clinic can be a valuable add-on. However, more trials should be performed to establish the value of eHealth in the follow-up of patients with AMI.

The median age of participants in our RCT was 59.7 years, which is slightly younger than the median age of the general population of patients with AMI at Leiden University Medical Center (63 years).^10^ This difference might be explained by the fact that more elderly patients rejected trial participation. However, it has been demonstrated that patients participating in RCTs are, in general, younger and have a lower a priori chance of death than nonparticipants.^18^ Therefore, it is unclear whether our patient population is younger because of the intervention or the randomized design of this study.

### Results of This Trial in Association With eHealth

This study was a feasibility RCT to evaluate the effect of smart technology on patients after AMI. In the popular literature, eHealth is often discussed as a tool to increase quality and patient satisfaction of care, by focusing more on preventing disease (instead of treating it), helping to integrate care by easing communication among health care practitioners, reducing duplication of diagnostic testing, and having patients perform some of their own diagnostic tests instead of trained health care staff.^19^ Although these are rather general remarks, the results of this study support some of this theory: first, patients were able to measure their own BP, ECG, and weight and transfer the data to the hospital without the presence or assistance of trained health care staff. This enabled the replacement of 2 physical outpatient clinic visits with 2 digital outpatient clinic visits, which are time-saving for patients and can, therefore, increase satisfaction. This may help reduce societal costs, especially for patients who are working.

Second, patients were able to accurately measure and transfer BP, a single-lead ECG, and weight, which are important diagnostic results for patients after AMI. This may reduce time of trained health care staff. Furthermore, patients can more easily send in clinically relevant measures (ECG and BP) to the hospital if indicated (eg, in case of palpitations).

Third, patients indicated that they appreciated extra control from the hospital, as well as the possibility to view their own health data. These are important secondary outcomes that indirectly support the theory that eHealth might improve patient involvement in clinical care. These results should, therefore, be corroborated in future studies.

### Limitations

This was a feasibility RCT to evaluate the effects of implementing eHealth in regular care. As such, some design choices were made that might have influenced the course of the trial. First, it was decided that every patient should receive the same smart technology intervention (weight scale, BP monitor, ECG device, and step counter). Every patient was instructed to use the same measurement frequency. This might have influenced measurement adherence and dropout rates in the intervention group, although a certain dropout percentage is frequently observed in RCTs in general. We recognize that this dropout might have influenced patient satisfaction rates, because patients who are not satisfied are inherently more likely to drop out. Therefore, patient satisfaction rates should be corroborated in future studies.

## Conclusions

Follow-up using smart technology for patients with AMI did not yield different percentages of regulated BP compared with patients who received standard care. This trial shows that smart technology and e-visits are feasible to implement in the follow-up of low-risk patients after AMI. Patient satisfaction and clinical outcomes in this instance were similar. Future research should corroborate these findings and should be performed to further define subgroups most likely to benefit from smartphone technology and to customize the smart technology intervention to an individual patient’s specific needs.

## Notes

Supplement 1. **Trial Protocol**

Supplement 2. **eAppendix. **Definitions of Adverse Cardiac Events **eReference.**

Supplement 3. **Data Sharing Statement**
