# Supplementary material for: Effect of Smartphone-Enabled Health Monitoring Devices vs Regular Follow-up on Blood Pressure Control Among Patients After Myocardial Infarction: A Randomized Clinical Trial
Source: JAMA Netw Open. 2020 Apr 16;3(4):e202165. doi: 10.1001/jamanetworkopen.2020.2165 (PMC7163406; doi:10.1001/jamanetworkopen.2020.2165)
Supplement: Supplement 1. — Trial Protocol [file jamanetwopen-3-e202165-s001.pdf]

The Box: using smart technology to improve one-year outcome of myocardial infarction patients

Version February 2017

- Correction April 2014: section 11.5: The sponsor/investigator has a liability insurance which is in accordance with article 7, subsection ~~6~~ 9 of the WMO

PROTOCOL TITLE 'The Box: using smart technology to improve one-year outcome of myocardial infarction patients'

**Protocol ID**	**The Box**
**Short title**	**The Box**
**EudraCT number**	***Not applicable***
**Version**	**2**
**Date**	**06-02-2017**
**Coordinating investigator/project leader**	***Professor M.J. Schalij, Cardiologist***<br>***M.J.Schalij@lumc.nl***<br>***071 526 2020***
**Principal investigator(s) (in Dutch: hoofdonderzoeker/ uitvoerder)**	***Professor M.J. Schalij, Cardiologist***<br>***M.J.Schalij@lumc.nl***<br>***071 526 2020***
**Sponsor (in Dutch: verrichter/opdrachtgever)**	***Department of Cardiology, Leiden University Medical Center***
**Subsidising party**	***Not applicable***
**Independent expert (s)**	**Dr. M. Bootsma, Cardiologist**<br>**Department of Cardiology**<br>**Leiden University Medical Center**<br>**m.bootsma@lumc.nl**
**Laboratory sites**	***Not applicable***
**Pharmacy**	***Not applicable***

PROTOCOL SIGNATURE SHEET

Name	Signature	Date
**Head of Department:**<br>***Professor M.J. Schalij, Cardiologist***		
**[Coordinating Investigator/Project leader/Principal Investigator]:**<br>***Professor M.J. Schalij, Cardiologist***		

TABLE OF CONTENTS

1. INTRODUCTION AND RATIONALE 8
2. OBJECTIVES 9
3. STUDY DESIGN 10
4. STUDY POPULATION 11
   4.1 Population (base) 11
   4.2 Inclusion criteria 11
   4.3 Exclusion criteria 11
   4.4 Sample size calculation 11
5. TREATMENT OF SUBJECTS 12
   5.1 Investigational product/treatment 12
   5.2 Use of co-intervention (if applicable) 14
   5.3 Escape medication (if applicable) 14
6. INVESTIGATIONAL PRODUCT 15
   6.1 Name and description of investigational product(s) 15
   6.2 Summary of findings from non-clinical studies .... **Error! Bookmark not defined.**
   6.3 Summary of findings from clinical studies 17
   6.4 Summary of known and potential risks and benefits 17
   6.5 Description and justification of route of administration and dosage 17
   6.6 Dosages, dosage modifications and method of administration 17
   6.7 Preparation and labelling of Investigational Medicinal Product 17
   6.8 Drug accountability 17
7. NON-INVESTIGATIONAL PRODUCT 18
   7.1 Name and description of non-investigational product(s) 18
   7.2 Summary of findings from non-clinical studies 18
   7.3 Summary of findings from clinical studies 18
   7.4 Summary of known and potential risks and benefits 18
   7.5 Description and justification of route of administration and dosage 18
   7.6 Dosages, dosage modifications and method of administration 18
   7.7 Preparation and labelling of Non Investigational Medicinal Product 18
   7.8 Drug accountability 18
8. METHODS 19
   8.1 Study parameters/endpoints 19
      8.1.1 Main study parameter/endpoint 19
      8.1.2 Secondary study parameters/endpoints (if applicable) 19
      8.1.3 Other study parameters (if applicable) 19
   8.2 Randomisation, blinding and treatment allocation 20
   8.3 Study procedures 20
   8.4 Withdrawal of individual subjects 21
      8.4.1 Specific criteria for withdrawal (if applicable) 21
   8.5 Replacement of individual subjects after withdrawal 21
   8.6 Follow-up of subjects withdrawn from treatment 21

8.7 Premature termination of the study 21
9. SAFETY REPORTING 22
9.1 Section 10 WMO event 22
9.2 AEs, SAEs and SUSARs 22
9.2.1 Adverse events (AEs) 22
9.2.2 Serious adverse events (SAEs) 22
9.2.3 Suspected unexpected serious adverse reactions (SUSARs) 23
9.3 Annual safety report 23
9.4 Follow-up of adverse events 23
9.5 [Data Safety Monitoring Board (DSMB) / Safety Committee] 23
10. STATISTICAL ANALYSIS 24
10.1 Primary study parameter(s) 24
10.2 Secondary study parameter(s) 24
10.3 Other study parameters 24
10.4 Interim analysis (if applicable) 24
11. ETHICAL CONSIDERATIONS 25
11.1 Regulation statement 25
11.2 Recruitment and consent 25
11.3 Objection by minors or incapacitated subjects (if applicable) 25
11.4 Benefits and risks assessment, group relatedness 25
11.5 Compensation for injury 25
11.6 Incentives (if applicable) 26
12. ADMINISTRATIVE ASPECTS, MONITORING AND PUBLICATION 27
12.1 Handling and storage of data and documents 27
12.2 Monitoring and Quality Assurance 27
12.3 Amendments 27
12.4 Annual progress report 27
12.5 End of study report 27
12.6 Public disclosure and publication policy 28
13. STRUCTURED RISK ANALYSIS 29
13.1 Potential issues of concern 29
13.2 Synthesis 29
14. REFERENCES 29

LIST OF ABBREVIATIONS AND RELEVANT DEFINITIONS

ABR	ABR form, General Assessment and Registration form, is the application form that is required for submission to the accredited Ethics Committee (In Dutch, ABR = Algemene Beoordeling en Registratie)
AE	Adverse Event
AR	Adverse Reaction
CA	Competent Authority
CCMO	Central Committee on Research Involving Human Subjects; in Dutch: Centrale Commissie Mensgebonden Onderzoek
CV	Curriculum Vitae
DSMB	Data Safety Monitoring Board
EU	European Union
EudraCT	European drug regulatory affairs Clinical Trials
GCP	Good Clinical Practice
IB	Investigator’s Brochure
IC	Informed Consent
IMP	Investigational Medicinal Product
IMPD	Investigational Medicinal Product Dossier
METC	Medical research ethics committee (MREC); in Dutch: medisch ethische toetsing commissie (METC)
(S)AE	(Serious) Adverse Event
SPC	Summary of Product Characteristics (in Dutch: officiële productinfomatie IB1-tekst)
Sponsor	The sponsor is the party that commissions the organisation or performance of the research, for example a pharmaceutical company, academic hospital, scientific organisation or investigator. A party that provides funding for a study but does not commission it is not regarded as the sponsor, but referred to as a subsidising party.
SUSAR	Suspected Unexpected Serious Adverse Reaction
Wbp	Personal Data Protection Act (in Dutch: Wet Bescherming Persoonsgevens)
WMO	Medical Research Involving Human Subjects Act (in Dutch: Wet Medisch-wetenschappelijk Onderzoek met Mensen

SUMMARY

Rationale: Smart technology could improve quality of care in patients after acute myocardial infarction (AMI) with either ST or non-ST elevation.
Objective: The objective of this study is to measure the effect of a smart technology intervention on patients after AMI.
Study design: The design of the study is a single-center, open randomized-controlled trial.
Study population: The study population consists of patients who have been discharged from the ward of the cardiology department of the Leiden University Medical Center after primary PCI for either ST or non-ST elevation myocardial infarction.
Intervention: Patients will be randomized to either “The Box” or regular follow-up. Patients who have been randomized to The Box will receive a box containing a smartphone compatible ECG monitor, a weight scale, an activity tracker and a blood pressure monitor. If patients are randomized to The Box, two of the four outpatient clinic visits will be replaced by an e-consult, in which a patient does not have to go to the hospital, but talks with his or her doctor or nurse practitioner via a secured video connection.
Main study parameters/endpoints: The primary endpoint of the study will be the percentage of patients with controlled blood pressure in both groups.
Nature and extent of the burden and risks associated with participation, benefit and group relatedness:
All devices used in this study are non-invasive, easy-to-use and electrically safe within its intended use. Using the devices is with very limited risks.
This study has some potential benefits for patients: first, patients can measure their own blood pressure, weight and activity, as well as record their own ECG. This can reassure patients and give them more insight in their own health (the so-called ‘patient empowerment’). Furthermore, more data gives the doctor more insight in the health of patients. This might lead to early detection of hypertension or arrhythmias such as atrial fibrillation. Lastly, due to the video connection system, patients do not have to come to the hospital, while receiving the same quality of care. A drawback is that patients have to measure their blood pressure, weight and ECG every day. Furthermore, patients have to fill in a couple of questionnaires, which will take some of their time.

1. INTRODUCTION AND RATIONALE

Over the past five years, smartphone compatible detectors of cardiovascular disease parameters have been released on the consumers market. Examples of these include heart rate monitors, ECG monitors, blood pressure monitors, activity trackers and fat percentages monitors. These monitors have often been validated and are CE-marked for use in the European Union within their intended use.(1)

Recent publications implicate that home monitoring with such consumer devices might improve quality of care. A study by Bosworth et al. in patients with hypertension showed that increased monitoring and subsequent treatment led to a better controlled blood pressure in patients who were treated for hypertension.(2)

It is well known that hypertension is not the only risk factor for cardiovascular disease, which can be monitored and adjusted by medication or lifestyle changes. Lack of physical activity and overweight are also well known risk factors.(3)

Therefore, we hypothesize that home monitoring of a combination of risk factors for cardiovascular disease improves quality of care in patients with acute myocardial infarction. To our knowledge, no study has yet investigated the clinical and cost-effectiveness in home monitoring in patients who have been discharged after acute myocardial infarction (with either ST- or non-ST-elevation).

Therefore, in this study we would like to investigate the clinical and cost-effectiveness of a smart technology intervention in patients who have been admitted to the Cardiac Care Unit of the Leiden University Medical Center with either ST- or non-ST elevation myocardial infarction.

2. OBJECTIVES

Primary Objective: the primary objective of this study is to investigate if a smart technology intervention could improve prognostic factors of coronary artery disease (blood pressure, cholesterol levels and physical activity)

Secondary Objectives:

1. Patient satisfaction of care in both groups
2. Establish total trial contact between hospital and patient (via phone, e-mail or face-to-face)
3. Total amount of hospital visits until one year after discharge
4. Quality of life in both groups
5. Overall mortality
6. Cardiac mortality
7. Major adverse cardiac events
    a. Cardiac death
    b. Reinfarction
    c. Heart Failure (chronic and acute)
    d. Revascularisation
    e. TIA
    f. Ischaemic stroke
8. Time between discharge from hospital and completion of device installation
9. Cost-effectiveness of interventions in both groups
10. Adherence to medication prescriptions

3. STUDY DESIGN

The design of the study is a single-center, open randomized-controlled trial. The study is estimated to take one and a half years (half year of patient inclusion and one year of follow-up).

4. STUDY POPULATION

4.1 Population (base)

Patients who have been admitted to the Cardiology department of the Leiden University Medical Center with an AMI (either ST-elevation of non-ST-elevation) will be asked to participate.

4.2 Inclusion criteria

In order to be eligible to participate in this study, a subject must meet all of the following criteria:

- Myocardial infarction (with either ST-elevation or non-ST elevation)
- Patient is able to communicate in English or Dutch language
- Patient is familiar with smartphone technology

4.3 Exclusion criteria

A potential subject who meets any of the following criteria will be excluded from participation in this study:

- Body Mass Index > 35 $kg \cdot m^{-2}$
- Patient is included in another randomized controlled trial
- Patient is <18 years old
- Patient is considered an incapacitated adult
- Patient is pregnant

4.4 Sample size calculation

A sample size was calculated using 'R'. ##Usage##
TwoSampleProportion.Equivalence(alpha, beta, p1, p2, k, delta, margin)

##Arguments##
alpha significance level
beta power = 1-beta
p1 the mean response rate for test drug
p2 the rate for reference drug
k k=n1/n2
delta delta=p1-p2
margin the superiority or non-inferiority margin

The following calculation was used:
a<-TwoSampleProportion.Equivalence(0.05,0.18,0.93,0.75,1,0.18,0.05)
print(a)
This yielded a sample size of 200 patients (100 patients per arm)

5. TREATMENT OF SUBJECTS

5.1 Investigational product/treatment

Patients who are randomized to “The Box” will get a Box, filled with several devices. These devices are listed below:

The AliveCor

The AliveCor is a handheld, easy-to-use, smartphone compatible device that can be attached to the smartphone. It has the size of a credit card. The AliveCor device is battery powered and electrically safe. The battery has to be changed one time in six months.

AliveCor measurement

The AliveCor produces a single lead ECG on the smartphone after 30 seconds of measurement. In order to make an ECG, patients have to sit down and relax their arms. When attaching fingers to the device for thirty seconds, a single-lead ECG can be made.

Storage of the ECGs

This AliveCor ECG is stored on the AliveCor app that can be downloaded on the smartphone.

All ECG’s, recorded by the AliveCor, will be send to AliveCor’s servers, which are located in Ireland.

Provider dashboard

All ECGs made by the AliveCor can be checked in the provider dashboard. The provider dashboard is AliveCor’s interface in which health care professionals can review a patient’s ECG. In order to do so, patients must have given the physician permission to review their ECGs! Without permission of the patient, an ECG cannot by reviewed.

In order to give the Leiden University Medical Center permission, an invitation is send by the primary investigator to a patients e-mail. By clicking the link and logging in with their AliveCor account, patients can give the Leiden University Medical Center permission.

Each patient which is randomized to the AliveCor will be instructed on how to use the AliveCor and how to give the LUMC permission to review the ECGs.

Taking an ECG with the AliveCor does not require any trained health care staff.

Withings Blood Pressure Monitor:

The Withings Blood Pressure Monitor is a Bluetooth enabled blood pressure cuff which can be placed around the upper arm of the patients. After activation in the Withings Health Mate app, Inflation and deflation of the cuff will lead to a systolic and diastolic blood pressure, as well as a heart rate, which are shown on the Withings Health Mate app on the smartphone. The Withings Blood Pressure Monitor is battery powered and electrically safe. It is CE-marked for blood pressure measurement.

Withings Weight Scale:

The Withings weight scale is a Bluetooth enabled weight scale which measures weight, body fat mass, heart rate and the CO_2 ppm in the air. It sends the results to the Withings Health Mate app. The Withings Weight Scale is battery powered and electrically safe. In order to measure weight, patients have to stand on the weight scale. Data are automatically send to the smartphone via Bluetooth.

Withings Activity Tracker:

The Withings Activity tracker is a Bluetooth enabled activity tracker which allows the user to count the steps taken a-day. The Withings activity tracker can be worn, to the user's preference, around the wrist or on the belt. The device is battery powered and electrically safe.

CardioSecur:

The CardioSecur is a battery powered and electrically safe device. It allows the user to make a 12-lead ECG with only four electrodes (Ambu P-00-S). The electrodes are placed in such a way that patients can attach the devices themselves, with no presence of trained health care staff needed. The CardioSecur app instructs the patients on how to attach the electrodes. Four gel based electrodes (Ambu P-00-S) are attached. One above the sternum, one under the sternum, one on the right side of the thorax and one on the left side of the thorax. The ECG will then appear on the patients smartphone.

Coding the data:

For the AliveCor app, the Withings app and the CardioSecur app, an e-mailaddress, first name, last name, birth date and password are required. Without those details, the app cannot be used. The investigators will therefore provide the users with an anonymous e-mailaddress. This is illustrated with a fictive patient:

Suppose the patients name is: Jan de Vries, birthdate 20-02-1948 and his e-mailaddress is jandevries@ziggo.nl.
The patient will register himself with a fake e-mailaddress. His personal details on the server will be:
TheBox A1234, birthdate 01-01-1950 and his e-mailaddress is theboxa1234@gmail.com
The patient can choose the password him/herself.

A spreadsheet will be made in which the codes and corresponding patient names are being kept. This document will be stored on the LUMC's servers and will be protected with a password. Only the study investigators will have access to the document.

5.2 Use of co-intervention

Not applicable

5.3 Escape medication

Not applicable

6. INVESTIGATIONAL PRODUCT

6.1 Name and description of investigational product(s)

Patients who are randomized to “The Box” will get a Box, filled with several devices. These devices are listed below:

<u>The AliveCor</u>

The AliveCor is a handheld, easy-to-use, smartphone compatible device that can be attached to the smartphone. It has the size of a credit card. The AliveCor device is battery powered and electrically safe. The battery has to be changed one time in six months.

AliveCor measurement

The AliveCor produces a single lead ECG on the smartphone after 30 seconds of measurement. In order to make an ECG, patients have to sit down and relax their arms. When attaching fingers to the device for thirty seconds, a single-lead ECG can be made.

Storage of the ECGs

This AliveCor ECG is stored on the AliveCor app that can be downloaded on the smartphone.

All ECG’s, recorded by the AliveCor, will be send to AliveCor’s servers, which are located in Ireland.

Provider dashboard

All ECGs made by the AliveCor can be checked in the provider dashboard. The provider dashboard is AliveCor’s interface in which health care professionals can review a patient’s ECG. In order to do so, patients must have given the physician permission to review their ECGs! Without permission of the patient, an ECG cannot by reviewed.

In order to give the Leiden University Medical Center permission, an invitation is send by the primary investigator to a patients e-mail. By clicking the link and logging in with their AliveCor account, patients can give the Leiden University Medical Center permission.

Each patient which is randomized to the AliveCor will be instructed on how to use the AliveCor and how to give the LUMC permission to review the ECGs.

Taking an ECG with the AliveCor does not require any trained health care staff.

<u>Withings Blood Pressure Monitor:</u>

The Withings Blood Pressure Monitor is a Bluetooth enabled blood pressure cuff which can be placed around the upper arm of the patients. After activation in the Withings Health Mate app, Inflation and deflation of the cuff will lead to a systolic and diastolic blood pressure, as well as a heart rate, which are shown on the Withings Health Mate app on the smartphone. The Withings Blood Pressure Monitor is battery powered and electrically safe. It is CE-marked for blood pressure measurement.

Withings Weight Scale:

The Withings weight scale is a Bluetooth enabled weight scale which measures weight, body fat mass, heart rate and the CO_2 ppm in the air. It sends the results to the Withings Health Mate app. The Withings Weight Scale is battery powered and electrically safe. In order to measure weight, patients have to stand on the weight scale. Data are automatically send to the smartphone via Bluetooth.

Withings Activity Tracker:

The Withings Activity tracker is a Bluetooth enabled activity tracker which allows the user to count the steps taken a-day. The Withings activity tracker can be worn, to the user's preference, around the wrist or on the belt. The device is battery powered and electrically safe.

CardioSecur:

The CardioSecur is a battery powered and electrically safe device. It allows the user to make a 12-lead ECG with only four electrodes (Ambu P-00-S). The electrodes are placed in such a way that patients can attach the devices themselves, with no presence of trained health care staff needed. The CardioSecur app instructs the patients on how to attach the electrodes. Four gel based electrodes (Ambu P-00-S) are attached. One above the sternum, one under the sternum, one on the right side of the thorax and one on the left side of the thorax. The ECG will then appear on the patients smartphone.

Coding the data:

For the AliveCor app, the Withings app and the CardioSecur app, an e-mailaddress, first name, last name, birth date and password are required. Without those details, the app cannot be used. The investigators will therefore provide the users with an anonymous e-mailaddress. This is illustrated with a fictive patient:

Suppose the patients name is: Jan de Vries, birthdate 20-02-1948 and his e-mailaddress is jandevries@ziggo.nl.

The patient will register himself with a fake e-mailaddress. His personal details on the server will be:
TheBox A1234, birthdate 01-01-1950 and his e-mailaddress is theboxa1234@gmail.com
The patient can choose the password him/herself.

A spreadsheet will be made in which the codes and corresponding patient names are being kept. This document will be stored on the LUMC's servers and will be protected with a password. Only the study investigators will have access to the document.

6.2 Summary of findings from clinical studies

Not applicable.

6.3 Summary of known and potential risks and benefits

All devices used in this study are non-invasive, easy-to-use and electrically safe. Using the devices is without any risks whatsoever. This study has some potential benefits for patients: first, patients can measure their own blood pressure, weight and activity, as well as record their own ECG. This can reassure patients and give them more insight in their own health (the so-called 'patient empowerment'). Furthermore, more data gives the doctor more insight in the health of patients. This might lead to early detection of hypertension or arrhythmias such as atrial fibrillation. Lastly, due to the video connection system, patients do not have to come to the hospital, while receiving the same quality of care, which will lessen the burden on patients.

6.4 Description and justification of route of administration and dosage

Not applicable

6.5 Dosages, dosage modifications and method of administration

Not applicable

6.6 Preparation and labelling of Investigational Medicinal Product

Not applicable

6.7 Drug accountability

Not applicable

7. NON-INVESTIGATIONAL PRODUCT

7.1 Name and description of non-investigational product(s)

Not applicable

7.2 Summary of findings from non-clinical studies

Not applicable

7.3 Summary of findings from clinical studies

Not applicable

7.4 Summary of known and potential risks and benefits

Not applicable

7.5 Description and justification of route of administration and dosage

Not applicable

7.6 Dosages, dosage modifications and method of administration

Not applicable

7.7 Preparation and labelling of Non Investigational Medicinal Product

Not applicable

7.8 Drug accountability

Not applicable

8. METHODS

8.1 Study parameters/endpoints

8.1.1 Main study parameter/endpoint

The primary endpoint of the study will be the percentage of patients with controlled blood pressure in both groups.

8.1.2 Secondary study parameters/endpoints

- Scores of SFQ-questionnaires
- Hospital-patient contact
- Amount of hospital visits
- Scores of Rand-36 questionnaires
- Major Adverse Cardiac Events
- Time between discharge from hospital and completion of device installation
- Cost-effectiveness of interventions in both groups
- Scores of medication-adherence questionnaires

8.1.3 Other study parameters (if applicable)

- Age
- Sex
- Highest educational level
- Employment (type of employment: farmer, industrial worker, office worker)
- Known heart conditions (arrhythmias, heart failure, etc)
- Alcohol intake
- Drug abuse
- Medications prior to onset symptoms of myocardial infarction
- Medications after discharge from the cardiology ward
- Time from onset symptoms to balloon
- Killip class
- Body Mass Index (BMI)
- Maximum troponin T
- Maximum CK-levels
- Previous MI
- Previous PCI
- Previous CABG
- Hypertension
- Hyperlipidaemia
- Current smoker
- Previous smoker (smoking cigarettes >1 month ago)
- Diabetes mellitus
- Dyslipidemia
- Signs of heart failure during admission

- Culprit coronary artery
- Dog owner

8.2 Randomisation, blinding and treatment allocation

Patients will be 1:1 randomized between "The Box" (intervention group) or regular follow-up (control group) after myocardial infarction. Randomization will be stratified per age. Block randomization will be performed (per 10 participants). A website (www.randomizer.org) will be used to generate lists, which will be kept a password protected spreadsheet stored on LUMC servers.

8.3 Study procedures

Directly after randomization, patients in the intervention group, will receive a box ("The Box") with all the devices described above. A dedicated PhD-Student will give instructions about the installation and usage of the devices. Furthermore, instruction videos have been made and are available on YouTube. Lastly, one week after discharge, patients will receive a phone call from a dedicated PhD-Student controlling if all devices have been installed.

Patients will be asked to measure their weight and blood pressure once a day. Furthermore, patients will be asked to record an ECG using the AliveCor once a day, preferably with 24 hours in between each ECG. Moreover, they are asked to record an ECG in case of any complaints of possible cardiac origin, as judged by the patient. Lastly, patients are asked to record an ECG with the CardioSecur once a week, preferably with 7 days in between each ECG. Furthermore, patients are asked to record an ECG in case the AliveCor shows "possible atrial fibrillation" as diagnosis.

All data will be automatically transferred to the Leiden University Medical Center. Patients do not have to actively send their data either via e-mail or any other medium. In order to code the data, patients will receive an anonymous e-mailaddress.

Lastly, two of the four outpatient clinical visits will be done via a video connection. Patients do not have to come to the hospital, but will be talking to the doctor via a secured videoconferencing connection. The content of the interview will be exactly the same as the content of a regular outpatient clinic visit. The same doctors will do the outpatient clinic visits as the digital outpatient clinic visits.

Patients who are randomized to the control group will receive regular care.

All patients will be asked to fill-in the Rand-36 questionnaire, the patient satisfaction questionnaire, the medication adherence questionnaire and the iPAQ questionnaire at 1 month, 6 months and 12 months after myocardial infarction. At baseline, patients will be asked to fill in an additional Rand-36 questionnaire. After 12 months, patients will be asked to fill in a costs-questionnaire. This cost questionnaire asks them how many times they have been at the GPs office, in a hospital or in physiotherapy sessions. Furthermore, it asks them if they have taken time of (unpaid) work. .

Lastly, after 12-months, patients will be asked to share their data of their pharmacy, in order for the investigators to see what medication they picked-up and how many pills

they have taken up at the pharmacy after one year. Furthermore, if patients have been admitted to another hospital, they will be asked permission to share these data with the investigators of the LUMC.

8.4 Withdrawal of individual subjects

Subjects can leave the study at any time for any reason if they wish to do so without any consequences. The investigator can decide to withdraw a subject from the study for urgent medical reasons.

8.4.1 Specific criteria for withdrawal

Not applicable

8.5 Replacement of individual subjects after withdrawal

If subjects decide to withdraw from the study, they will not be replaced.

8.6 Follow-up of subjects withdrawn from treatment

Subjects who withdraw from treatment will be referred to regular follow-up.

8.7 Premature termination of the study

Not applicable

9. SAFETY REPORTING

9.1 Section 10 WMO event

In accordance to section 10, subsection 1, of the WMO, the investigator will inform the subjects and the reviewing accredited METC if anything occurs, on the basis of which it appears that the disadvantages of participation may be significantly greater than was foreseen in the research proposal. The study will be suspended pending further review by the accredited METC, except insofar as suspension would jeopardise the subjects' health. The investigator will take care that all subjects are kept informed.

9.2 AEs, SAEs and SUSARs

9.2.1 Adverse events (AEs)

Adverse events are defined as any undesirable experience occurring to a subject during the study, whether or not considered related to the investigational product. All adverse events reported spontaneously by the subject or observed by the investigator or his staff will be recorded.

9.2.2 Serious adverse events (SAEs)

A serious adverse event is any untoward medical occurrence or effect that at any dose:

- results in death;
- is life threatening (at the time of the event);
- requires hospitalisation or prolongation of existing inpatients' hospitalisation;
- results in persistent or significant disability or incapacity;
- is a congenital anomaly or birth defect;
- Any other important medical event that may not result in death, be life threatening, or require hospitalization, may be considered a serious adverse experience when, based upon appropriate medical judgement, the event may jeopardize the subject or may require an intervention to prevent one of the outcomes listed above.

The sponsor will report the SAEs through the web portal *ToetsingOnline* to the accredited METC that approved the protocol, within 15 days after the sponsor has first knowledge of the serious adverse events.

SAEs that result in death or are life threatening should be reported expedited. The expedited reporting will occur not later than 7 days after the responsible investigator

has first knowledge of the adverse event. This is for a preliminary report with another 8 days for completion of the report.

Because this study is without any risks for the patient, we kindly ask the committee to permit us to report all SAEs once per year.

9.2.3 Suspected unexpected serious adverse reactions (SUSARs)

Not applicable

9.3 Annual safety report

Not applicable

9.4 Follow-up of adverse events

All AEs will be followed until they have abated, or until a stable situation has been reached. Depending on the event, follow up may require additional tests or medical procedures as indicated, and/or referral to the general physician or a medical specialist. SAEs need to be reported till end of study within the Netherlands, as defined in the protocol

9.5 [Data Safety Monitoring Board (DSMB) / Safety Committee]

Not applicable

10. STATISTICAL ANALYSIS

10.1 Primary study parameter(s)

To compare the two percentages, a Chi-Squared test in SPSS will be done.

10.2 Secondary study parameter(s)

Scores of questionnaires will be compared using a Chi-Squared test. Normally distributed data will be compared using a student t test.

10.3 Other study parameters

Other study parameters are only obtained from patients or patient information records to give inside on differences of the two groups on potential confounders. As any differences will be random, no statistics will be done on this.

10.4 Interim analysis (if applicable)

Not applicable

11. ETHICAL CONSIDERATIONS

11.1 Regulation statement

The study will be conducted according to the principles of the Declaration of Helsinki (version 10, October 2013) and in accordance with the Medical Research Involving Human Subjects Act (WMO).

11.2 Recruitment and consent

Patients will be asked to participate by a doctor or nurse practitioner or dedicated PhD-Student if they are admitted. Patients will be given written information about the study. Patients are allowed a reflection period if they wish to have so. Patients will have to decide to participate before discharge from the hospital, in order to prevent unnecessary hospital visits for the patient. If patients agree to participate, written informed consent will be obtained by a dedicated PhD-Student.

Of course, patients can refuse participation at any time without having to give a reason.

11.3 Objection by minors or incapacitated subjects (if applicable)

Not applicable

11.4 Benefits and risks assessment, group relatedness

All devices used in this study are non-invasive, easy-to-use and electrically safe. Using the devices is without any risks whatsoever. This study has some potential benefits for patients: first, patients can measure their own blood pressure, weight and activity, as well as record their own ECG. This can reassure patients and give them more insight in their own health (the so-called ‘patient empowerment’). Furthermore, more data gives the doctor more insight in the health of patients. This might lead to early detection of hypertension or arrhythmias such as atrial fibrillation. Lastly, due to the video connection system, patients do not have to come to the hospital, while receiving the same quality of care.

Summarizing, we presented a study with no risks and a couple of potential benefits for participating patients as well for future patients. We therefore believe this study is ethically justified.

11.5 Compensation for injury

As this study is without any risk for the patient whatsoever, we would like to obtain dispensation from the statutory obligation to provide insurance. A reasoned request has been included.

11.6 Incentives

All devices will be provided by the LUMC. Patients do not have to pay for the devices. Patients are allowed to keep their devices after the trial is finished. Patients who withdraw from the study are allowed to keep their devices as well.

12. ADMINISTRATIVE ASPECTS, MONITORING AND PUBLICATION

12.1 Handling and storage of data and documents

Data Collection Forms, Case Report Forms, Informed Consent Forms and all other study documentation containing subject information will be stored under locked conditions when not in use. Computers and all storage devices containing study data will be password-protected. Data stored on the computer will use an alphanumeric code to identify the subject. Access to data is restricted to study personnel and when required the REC or other regulatory bodies as required by law. Essential trial documents and data will be retained for at least five years.

12.2 Monitoring and Quality Assurance

Not applicable

12.3 Amendments

Amendments are changes made to the research after a favourable opinion by the accredited METC has been given. All amendments will be notified to the METC that gave a favourable opinion.

12.4 Annual progress report

The sponsor/investigator will submit a summary of the progress of the trial to the accredited METC once a year. Information will be provided on the date of inclusion of the first subject, numbers of subjects included and numbers of subjects that have completed the trial, serious adverse events/ serious adverse reactions, other problems, and amendments.

12.5 End of study report

The investigator will notify the accredited METC of the end of the study within a period of 8 weeks. The end of the study is defined as the last patient's last visit.

In case the study is ended prematurely, the investigator will notify the accredited METC within 15 days, including the reasons for the premature termination.

Within one year after the end of the study, the investigator/sponsor will submit a final study report with the results of the study, including any publications/abstracts of the study,

to the accredited METC.

12.6 Public disclosure and publication policy

The study is registered at the Dutch Trial Register (Nederlands Trial Register, NTR) and ClinicalTrials.gov. It is our intention to publish the results in peer reviewed medical journal.

13. STRUCTURED RISK ANALYSIS

13.1 Potential issues of concern

Not applicable

13.2 Synthesis

All devices used in this study are non-invasive, easy-to-use and electrically safe. Using the devices is without any risks whatsoever. This study has some potential benefits for patients: first, patients can measure their own blood pressure, weight and activity, as well as record their own ECG. This can reassure patients and give them more insight in their own health (the so-called ‘patient empowerment’). Furthermore, more data gives the doctor more insight in the health of patients. This might lead to early detection of hypertension or arrhythmias such as atrial fibrillation. Lastly, due to the video connection system, patients do not have to come to the hospital, while receiving the same quality of care.

Summarizing, we presented a study with no risks and a couple of potential benefits for participating patients as well for future patients. We therefore believe this study is ethically justified.

14. REFERENCES

Reference List

(1) Withings. Available via: www.withings.com. 2016.

(2) Bosworth HB, Powers BJ, Olsen MK, McCant F, Grubber J, Smith V, et al. Home blood pressure management and improved blood pressure control: results from a randomized controlled trial. Arch Intern Med 2011 Jul 11;171(13):1173-80.

(3) Perk J, De BG, Gohlke H, Graham I, Reiner Z, Verschuren M, et al. European Guidelines on cardiovascular disease prevention in clinical practice (version 2012). The Fifth Joint Task Force of the European Society of Cardiology and Other Societies on Cardiovascular Disease Prevention in Clinical Practice (constituted by representatives of nine societies and by invited experts). Eur Heart J 2012 Jul;33(13):1635-701.
