# Supplementary material for: Effect of Smartphone-Enabled Health Monitoring Devices vs Regular Follow-up on Blood Pressure Control Among Patients After Myocardial Infarction: A Randomized Clinical Trial
Source: JAMA Netw Open. 2020 Apr 16;3(4):e202165. doi: 10.1001/jamanetworkopen.2020.2165 (PMC7163406; doi:10.1001/jamanetworkopen.2020.2165)
Supplement: Supplement 2. — eAppendix. Definitions of Adverse Cardiac Events eReference. [file jamanetwopen-3-e202165-s002.pdf]

# Supplementary Online Content

Treskes RW, van Winden LAM, van Keulen N, et al. Effect of smartphone-enabled health monitoring devices vs regular follow-up on blood pressure control among patients after myocardial infarction: a randomized clinical trial. *JAMA Netw Open*. 2020;3(4):e202165. doi:10.1001/jamanetworkopen.2020.2165

**eAppendix.** Definitions of Adverse Cardiac Events

**eReference**

This supplementary material has been provided by the authors to give readers additional information about their work.

© 2020 Treskes RW et al. *JAMA Network Open.*

## eAppendix. Definitions of Adverse Cardiac Events

- Hospitalization: a hospitalization was defined as an admission to the department of cardiology of the Leiden University Medical Center which included a date change.
- Recurrent AMI: in order for an event to be classified as a recurrent AMI, patients needed to have symptoms suggestive of AMI (notably chest pain, radiation to cheeks or left arm), ST-T deviations from the ECG at discharge and two elevated troponin levels, not being measured after elective percutaneous coronary intervention. Silent MIs were therefore not registered in this trial.[1]
- HF event: a HF event was defined as a "hospitalization with a primary diagnosis of HF"[1], for which "the patient is required to have typical signs, symptoms and diagnostic testing results consistent with the diagnosis of HF."[1]
- Out-of-hospital cardiac arrest (OHCA): an OHCA was defined as a sudden loss of consciousness, primary caused by ventricular tachycardia, ventricular fibrillation, asystole or pulseless electrical activity (as assessed by trained medical staff, e.g. ambulance staff) for which cardiopulmonary resuscitation was performed.
- Elective revascularization: Elective revascularization is defined as a percutaneous coronary intervention with placement of a drug eluting stent or bare metal stent that resulted from an electively planned coronary angiogram.

The definitions of hospitalization, recurrent AMI and HF were in accordance with the "2017 Cardiovascular and Stroke Endpoint Definitions for Clinical Trials".[1]

## eReference

1. Hicks KA, Mahaffey KW, Mehran R, et al. 2017 Cardiovascular and Stroke Endpoint Definitions for Clinical Trials. *Circulation.* 2018;137(9):961-972.

© 2020 Treskes RW et al. *JAMA Network Open.*
