# Supplementary material for: Effect of Smartphone-Enabled Health Monitoring Devices vs Regular Follow-up on Blood Pressure Control Among Patients After Myocardial Infarction: A Randomized Clinical Trial
Source: JAMA Netw Open. 2020 Apr 16;3(4):e202165. doi: 10.1001/jamanetworkopen.2020.2165 (PMC7163406; doi:10.1001/jamanetworkopen.2020.2165)
Supplement: Supplement 3. — Data Sharing Statement [file jamanetwopen-3-e202165-s003.pdf]

Data Sharing Statement

Treskes. Effect of Smartphone-Enabled Health Monitoring Devices vs Regular Follow-up on Blood Pressure Control Among Patients After Myocardial Infarction. *JAMA Netw Open*. Published April 16, 2020. 10.1001/jamanetworkopen.2020.2165

Data
Data available: No

Additional Information
Explanation for why data not available: The European Privacy Law doesn't allow us to make data obtained for research publicly available
